# Predictive value of left atrial diameter and epicardial adipose tissue in sleep apnea and heart failure with preserved ejection fraction

**DOI:** 10.3389/fcvm.2026.1787318

**Published:** 2026-07-03

**Authors:** Gong Yuqianhui, Liu Yuanya, Zhang Hao, Wen Zhanpeng, Quan Yong, Feng Li, Huang Xuansheng, Sun Yanxiang

**Affiliations:** Department of Cardiovascular Medicine, Zhongshan City People’s Hospital, Zhongshan, China

**Keywords:** epicardial adipose tissue, heart failure with preserved ejection fraction, left atrial diameter, risk factors, sleep apnea

## Abstract

**Objective:**

This study aims to investigate the association of left atrial diameter (LAD) and epicardial adipose tissue (EAT) with heart failure with preserved ejection fraction (HFpEF) in patients with sleep apnea (SA) and to evaluate their potential as markers for risk stratification.

**Methods:**

A total of 170 hospitalized patients with SA, diagnosed by polysomnography and who underwent cardiac CT between July 2022 and June 2023, were enrolled. Participants were divided into an HFpEF group (*n* = 96) and a non-HFpEF group (*n* = 74). Clinical data, including EAT relative volume and echocardiographic parameters, were collected and compared. Logistic regression and ROC curve analyses were used to assess factors associated with HFpEF and their discriminatory ability, and Cox regression was performed for prognostic evaluation.

**Results:**

Patients with HFpEF had significantly higher LAD, apnea–hypopnea index, EAT relative volume, and NT-proBNP levels than those without HFpEF (all *P* < 0.05). Multivariate logistic regression showed that female sex, NT-proBNP level, LAD, and EAT relative volume were independently associated with HFpEF in SA patients. A combined model incorporating these four factors yielded an area under the curve of 0.971 (95% CI: 0.942–1.000) for discriminating HFpEF. Cox regression analysis revealed that NT-proBNP was independently associated with poor prognosis among SA patients with HFpEF.

**Conclusion:**

LAD was positively correlated with EAT relative volume, AHI, and NT-proBNP levels. Both LAD and EAT relative volume were significantly associated with HFpEF in SA patients and may serve as potential markers for identifying high-risk individuals.

## 1 Introduction

Heart failure is a complex clinical syndrome caused by structural and/or functional abnormalities of the heart, resulting in impaired ventricular filling and/or ejection capacity with corresponding clinical manifestations (1). Among these, patients with heart failure with preserved ejection fraction (HFpEF) exhibit increased left ventricular (LV) stiffness, which is closely associated with left atrial function. The left atrium (LA) plays a pivotal role in regulating left ventricular filling and maintaining cardiovascular function by retaining pulmonary venous return and facilitating ventricular filling (2). Left atrial enlargement has been identified as a predictor of adverse outcomes in multiple cardiovascular conditions, including heart failure, atrial fibrillation, and cardiovascular mortality (2, 3). Furthermore, sleep apnea (SA) commonly coexists with heart failure, with obstructive sleep apnea (OSA) being one of the predominant subtypes. OSA promotes intermittent hypoxia and activates the sympathetic nervous system and the renin–angiotensin–aldosterone system, leading to systemic inflammatory and oxidative stress. This impairs left ventricular diastolic function and contributes to the onset and progression of HFpEF (4). Previous studies have revealed that 80% of HFpEF patients exhibit significant sleep apnea, with 62% presenting OSA (5). Epicardial adipose tissue (EAT), primarily composed of white adipose tissue, correlates directly with systemic and visceral fat content. Excessive accumulation of adipose tissue in specific sites, such as visceral fat and EAT, can function as a metabolically active endocrine organ, promoting inflammation and potentially contributing to cardiac and vascular remodeling and dysfunction (6). It represents one of the most significant risk factors for atherosclerosis and adverse cardiovascular events, emerging as a novel therapeutic target in cardiovascular disease (7). This study aims to evaluate the relationships among LAD, EAT, and SA and to assess their predictive value for the onset and prognosis of HFpEF, thereby providing new insights for the clinical management of HFpEF.

## 2 Information and methods

### 2.1 Study participants

**Figure 1:**
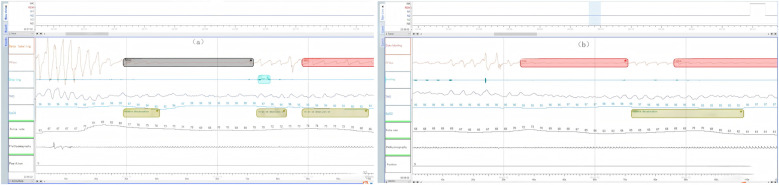
Polysomnography (PSG) tracings of typical respiratory events. **(a)** Mixed sleep apnea (MSA) accompanied by obstructive sleep apnea (OSA). The gray box marks an MSA episode, and red boxes indicate OSA episodes. **(b)** Isolated obstructive sleep apnea (OSA), with two OSA events highlighted by red boxes. PFlow, pressure flow; THO, thoracic respiratory effort; SpO₂, peripheral oxygen saturation.

Patients hospitalized in the Department of Cardiovascular Medicine at Zhongshan City People's Hospital from July 2022 to June 2023 were eligible for inclusion if they met the following criteria: (1) age 18–80 years; (2) SA diagnosis confirmed by polysomnography (PSG), with an apnea–hypopnea index (AHI) ≥ 5 events/h (Figures 1a, b) (8); and (3) availability of echocardiographic and cardiac CT data, detailed medical records, laboratory data, and signed informed consent. Patients were excluded if they had chronic cor pulmonale, congenital heart disease, moderate-to-severe valvular heart disease, inherited cardiomyopathy, acute myocarditis, malignancy, severe liver dysfunction (Child–Pugh score ≥ 10), or severe renal impairment (eGFR < 30 mL/min/1.73 m^2^ or requiring dialysis). Patients were classified into HFpEF and non-HFpEF groups according to the *National Heart Failure Guidelines 2023* (1). The diagnosis of HFpEF was established based on a multiparametric clinical assessment and required the fulfillment of the following criteria: (1) the presence of symptoms and/or signs of heart failure; (2) a preserved left ventricular ejection fraction (LVEF) ≥ 50%; and (3) elevated NT-proBNP levels, supplemented by echocardiographic evidence of structural heart disease or diastolic dysfunction (e.g., E/A ratio). The non-HFpEF group included patients with sleep apnea who did not meet the diagnostic criteria for HFpEF. Although some patients in this group exhibited mildly elevated NT-proBNP levels, they did not present with clinical symptoms of heart failure or exhibit echocardiographic evidence sufficient to fulfill HFpEF diagnostic criteria.

Furthermore, none of the patients received continuous positive airway pressure (CPAP) therapy during the study period, thereby eliminating the potential confounding effects of CPAP on cardiac remodeling and clinical outcomes.

### 2.2 Collection of general information

**Figure 2:**
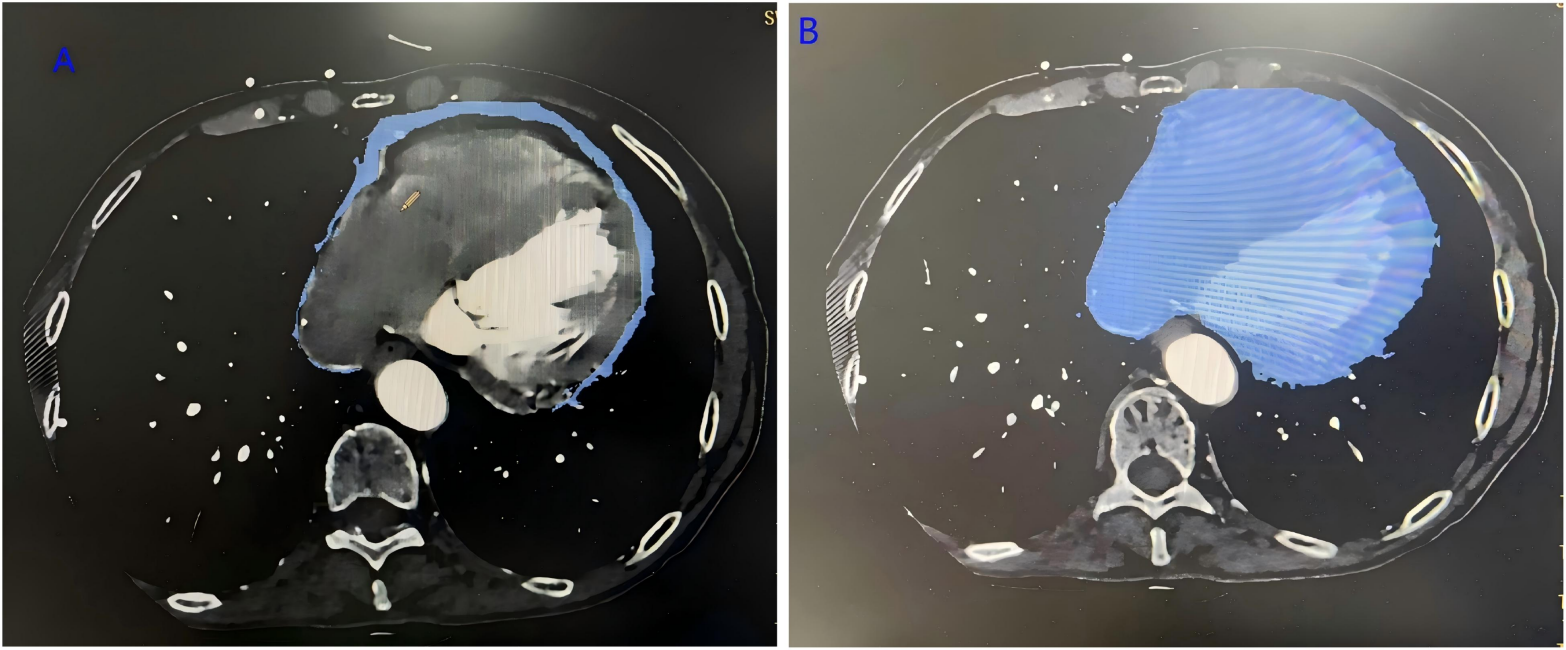
Cardiac CT images illustrating semi-automated segmentation and quantification of epicardial adipose tissue (EAT). **(A)** Thin-slice manual contour delineation along the pericardial margin to outline EAT; **(B)** Fully filled color-coded EAT mask reconstructed by post-processing software for EAT volume calculation.

General data of patients were collected, including age, height, weight, and body mass index (BMI). Blood biochemical indicators included serum creatinine, triglycerides (TGs), total cholesterol (TC), low-density lipoprotein cholesterol (LDL-C), fasting blood glucose, N-terminal pro-B-type natriuretic peptide (NT-proBNP), interleukin-6, and nuclear factor kappa-B (NF-κB). Imaging examinations included echocardiography to measure left atrial diameter (LAD), left ventricular end-diastolic diameter (LVEDD), LVEF, and interventricular septal thickness (IVST) and cardiac CT (Figures 2A, B) to measure EAT volume (mL) (9). EAT relative volume = EAT volume (mL)/body surface area (m^2^). Patients were followed for 1 year after discharge, and the endpoint event was all-cause death or hospitalization.

### 2.3 Statistical analysis

Data were analyzed using SPSS version 26.0. Continuous variables were expressed as mean ± SD or median (interquartile range). Categorical variables were presented as numbers (percentages). Between-group comparisons were conducted using the independent-samples *t*-test, Mann–Whitney *U*-test, or chi-square test, as appropriate. Correlations were assessed using Pearson or Spearman tests. Multivariate logistic regression was used to identify risk factors associated with HFpEF in patients with SA. The predictive performance was evaluated using receiver operating characteristic (ROC) curves and area under the curve (AUC). Prognostic factors were assessed using Cox regression. A two-tailed *P* < 0.05 was considered statistically significant.

## 3 Results

### 3.1 Comparison of general clinical data

A total of 170 SA patients were included, of whom 96 had HFpEF and 74 did not. Compared with the non-HFpEF group, HFpEF patients were older, had a higher proportion of women, and exhibited higher BMI, LAD, AHI, EAT relative volume, and NT-proBNP levels (all *P* < 0.05). No significant differences were observed between the groups in blood pressure, comorbidities (hypertension, diabetes, smoking, alcohol), laboratory biomarkers (IL-6, NF-κB, lipids, glucose), or echocardiographic parameters (LVEF, LVEDD, IVST) (all *P* > 0.05) (Table 1).

**Table 1:** Comparison of baseline characteristics between SA patients with and without HFpEF.

Group	Control (*N* = 74)	HFpEF (*N* = 96)	*P* value
Age, years	58 (49, 65)	69 (63, 74)	<0.001
Female, *n* (%)	24 (31.6)	61 (63.5)	<0.001
BMI, kg/m^2^	23.4 (21.1, 26.8)	26.1 (23.1, 29.0)	<0.001
SBP, mmHg	125 (116, 141)	129 (120, 141)	0.527
DBP, mmHg	82 ± 14	80 ± 13	0.323
Diabetes, *n* (%)	9 (11.8)	14 (14.6)	0.600
Hypertension, *n* (%)	31 (40.8)	53 (55.2)	0.060
Smoking, *n* (%)	15 (19.7)	12 (12.5)	0.195
Alcohol, *n* (%)	6 (7.9)	7 (7.3)	0.882
AHI, events/h	13.5 ± 3.6	16.7 ± 3.6	<0.001
NT-proBNP, pg/L	361 (291, 469)	906 (802, 1,063)	<0.001
IL-6, μmol/L	20 (9, 32)	21 (13, 35)	0.546
NF-κB, μmol/L	0.21 (0.09, 0.42)	0.30 (0.12, 0.38)	0.386
SCr, μmol/L	84 (65, 93)	77 (61, 91)	0.311
TC, mmol/L	4.62 (3.72, 5.40)	4.52 (3.48, 5.13)	0.302
TG, mmol/L	1.40 (0.94, 1.83)	1.24 (0.91, 1.79)	0.208
LDL-C, mmol/LL	2.83 (2.13, 3.65)	2.62 (1.89, 3.03)	0.052
FBG, mmol/L	5.26 (4.90, 5.91)	5.27 (4.82, 6.13)	0.999
LVEF, %	63 (41, 66)	62 (58, 66)	0.263
LVEDD, mm	46 (44, 51)	47 (45, 49)	0.729
IVST, mm	10.00 (9.00, 10.25)	10.00 (9.00, 11.00)	0.358
LAD, mm	32 ± 6	39 ± 6	<0.001
EAT relative volume, mL/m^2^	11.0 (7.75,15.00)	15.0 (11.0, 19.0)	<0.001

Data are presented as median (IQR), mean ± SD, or *n* (%). AHI, apnea–hypopnea index; BMI, body mass index; DBP, diastolic blood pressure; EAT, epicardial adipose tissue; HFpEF, heart failure with preserved ejection fraction; IL-6, interleukin-6; IVST, interventricular septal thickness; LAD, left atrial diameter; LDL-C, low-density lipoprotein cholesterol; LVEDD, left ventricular end-diastolic diameter; LVEF, left ventricular ejection fraction; NF-κB, nuclear factor kappa-B; NT-proBNP, N-terminal pro-B-type natriuretic peptide; SBP, systolic blood pressure.

*p* < 0.05 indicates a significant difference.

### 3.2 Correlation analysis of LAD and EAT relative volume, AHI, and other heart failure-related indices

Results showed that (1) in the overall population, LAD was positively correlated with BMI, history of hypertension, AHI, NT-proBNP level, serum creatinine, LVEDD, IVST, and relative EAT volume (all *P* < 0.05) and negatively correlated with female gender, TC, LDL-C, and LVEF (all *P* < 0.05). In patients with HFpEF, LAD showed positive correlations with BMI, history of hypertension, AHI, NT-proBNP level, serum creatinine, LVEDD, and relative EAT volume (all *P* < 0.05) and negative correlations with female gender, LDL-C, and LVEF (all *P* < 0.05) (Table 2). (2) In the overall population, relative EAT volume was positively correlated with age, diastolic blood pressure, AHI, NF-κB levels, TG, and LAD (all *P* < 0.05). In HFpEF patients, relative EAT volume was positively correlated with diastolic blood pressure, TG, and LAD (all *P* < 0.05) (Table 3).

**Table 2:** Results of the correlation analysis between left atrial anteroposterior diameter and other parameters in the general population and in patients with HFpEF.

Group	Overall population (*N* = 170)		HFpEF (*N* = 96)	
	*R*	*P*	*r*	*P*
EAT relative volume, mL/m^2^	0.256	0.004	0.306	0.008
AHI, events/h	0.245	0.003	0.263	0.016
NT-proBNP, pg/mL	0.522	0.000	0.383	0.000
Female	−0.187	0.014	−0.259	0.011
Age, years	0.086	0.263	0.148	0.150
BMI, kg/m^2^	0.307	0.000	0.305	0.003
LVEDD, mm	0.510	0.000	0.389	0.000
SBP, mmHg	−0.014	0.852	−0.069	0.502
DBP, mmHg	0.035	0.650	0.047	0.649
Hypertension, *n* (%)	0.193	0.011	0.276	0.006
Diabetes, *n* (%)	0.040	0.601	0.040	0.702
Smoking, *n* (%)	−0.074	0.332	−0.004	0.969
Alcoho, *n* (%)	−0.093	0.225	−0.037	0.721
SCr, μmol/L	0.233	0.002	0.206	0.044
IL-6, μmol/L	0.073	0.412	0.116	0.355
NF-κB, μmol/L	0.522	<0.001	0.383	<0.001
FBG, mmol/L	0.048	0.537	−0.058	0.576
TC, mmol/L	−0.238	0.002	−0.191	0.068
TG, mmol/L	0.007	0.928	0.030	0.773
LDL-C, mmol/L	−0.231	0.003	−0.247	0.018
IVST, mmol/L	0.174	0.023	0.088	0.393

**Table 3:** Results of the correlation analysis between EAT relative volume and other indicators in the general population and in patients with HFpEF.

Group	Overall population (*N* = 170)		HFpEF (*N* = 96)	
	*R*	*P*	*r*	*P*
LAD, mm	0.256	0.004	0.306	0.008
AHI, events/h	0.210	0.003	0.056	0.651
NT-proBNP, pg/mL	0.142	0.117	0.106	0.000
Age, years	0.364	0.001	0.147	0.211
Female	0.044	0.625	−0.064	0.587
BMI, kg/m^2^	0.170	0.058	0.158	0.179
LVEDD, mm	−0.026	0.771	−0.024	0.839
SBP, mmHg	0.141	0.115	0.103	0.385
DBP, mmHg	0.208	0.020	0.343	0.003
Hypertension, *n* (%)	0.014	0.876	−0.065	0.582
Diabetes, *n* (%)	0.030	0.736	−0.104	0.378
Smoking, *n* (%)	0.071	0.430	0.036	0.762
Alcohol, *n* (%)	0.036	0.688	0.031	0.794
SCr, μmol/L	−0.034	0.708	−0.065	0.583
IL-6, μmol/L	−0.006	0.957	−0.057	0.695
NF-κB, μmol/L	0.220	0.020	0.173	0.159
FBG, mmol/L	0.012	0.893	−0.056	0.642
TC, mmol/L	−0.121	0.183	−0.073	0.546
TG, mmol/L	0.201	0.026	0.334	0.004
LDL-C, mmol/L	−0.082	0.366	−0.055	0.651
IVST, mmol/L	0.066	0.462	−0.080	0.499

AHI, apnea–hypopnea index; BMI, body mass index; DBP, diastolic blood pressure; EAT, epicardial adipose tissue; HFpEF, heart failure with preserved ejection fraction; IL-6, interleukin-6; IVST, interventricular septal thickness; LAD, left atrial diameter; LDL-C, low-density lipoprotein cholesterol; LVEDD, left ventricular end-diastolic diameter; LVEF, left ventricular ejection fraction; NF-κB, nuclear factor kappa-B; NT-proBNP, N-terminal pro-B-type natriuretic peptide; SBP, systolic blood pressure.

### 3.3 Risk prediction of HFpEF in patients with sleep apnea by LAD and EAT

Among patients with SA (*n* = 170), using the presence of HFpEF as the dependent variable (*n* = 96), univariate binary logistic regression analysis showed that female sex, age, BMI, NT-proBNP level, LDL-C, LAD, and relative EAT volume were risk factors for HFpEF in patients with SA. Multivariate binary logistic regression analysis revealed that female sex, NT-proBNP level, LAD, and relative EAT volume were independent risk factors for HFpEF in patients with SA (Table 4).

**Table 4:** Risk prediction of HEFpEF in patients with sleep apnea caused by LAD and EAT (*n* = 170).

Group	Univariate logistic		Multivariate logistic	
	OR (95% CI)	*P*	OR (95% CI)	*P*
Gender	3.78 (2.00–7.15)	<0.001	4.70 (1.39–15.84)	0.012
Age, years	1.09 (1.051.12)	<0.001	1.04 (0.99–1.09)	0.168
BMII, kg/m^2^	1.11 (1.03–1.20)	0.006	1.01 (.897–1.14)	0.863
Log_10_ NT-proBNP	3.39 (1.83–6.30)	<0.001	7.62 (2.31–25.13)	<0.001
LDL-C, mmol/L	0.67 (0.48–0.94)	0.022	0.62 (0.34–1.13)	0.120
LAD, mm	1.21 (1.14–1.29)	<0.001	1.16 (1.04–1.28)	0.005
EAT relative volume, mL/m^2^	1.23 (1.14–1.32)	<0.001	1.15 (1.01–1.29)	0.025

BMI, body mass index; NT-proBNP, N-terminal brain natriuretic peptide precursor; LDL-C, low-density lipoprotein; LAD, left atrial diameter; EAT relative volume, epicardial adipose tissue relative volume.

### 3.4 Predictive value of LAD and EAT for the occurrence of HFpEF in patients with SA

**Figure 3:**
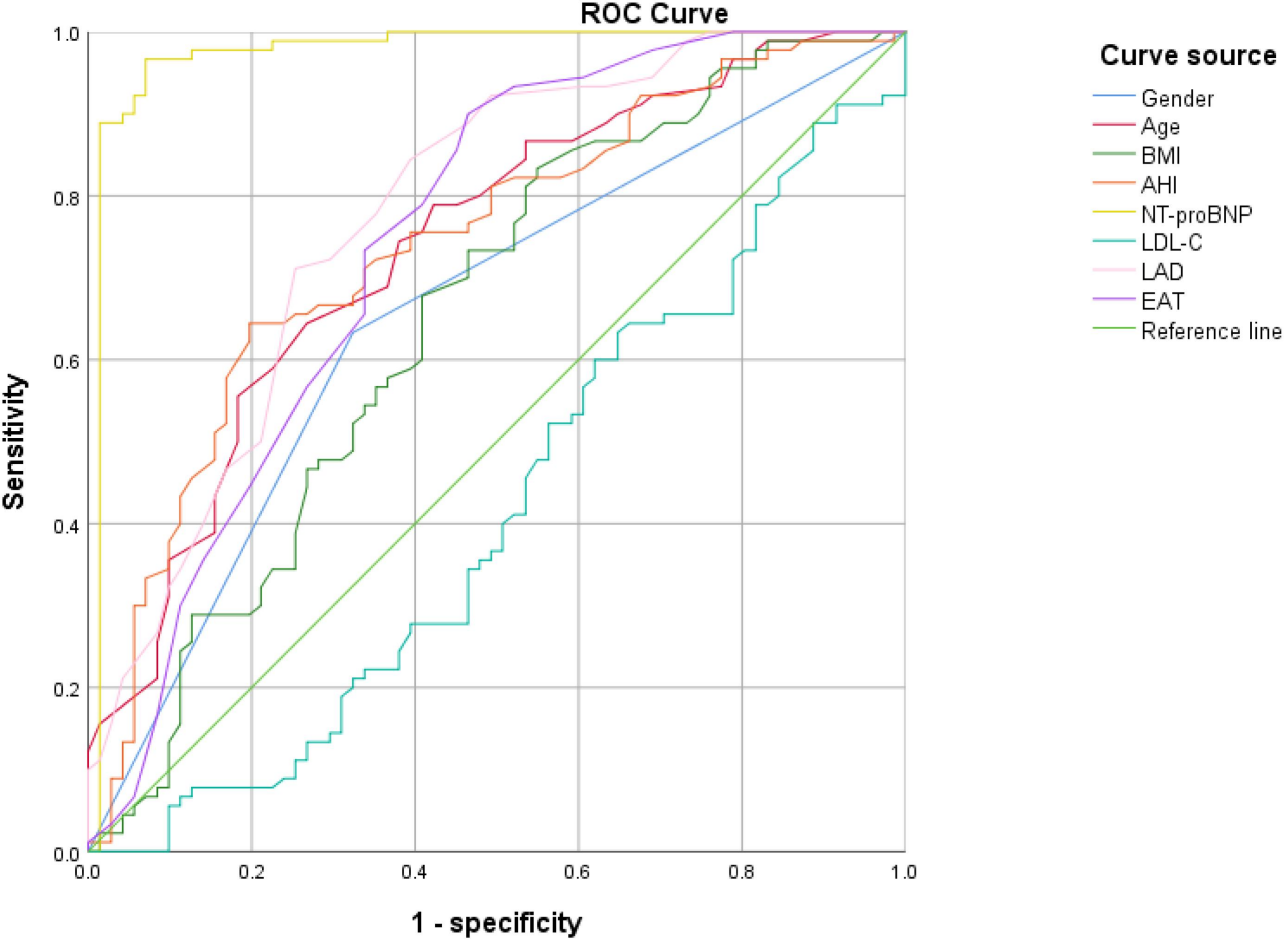
ROC curve analysis for the prediction of LAD, age, and EAT relative volume in patients with SA and HFpEF. BMI, body mass index; AHI, apnea–hypopnea index; BMI, body mass index; EAT, epicardial adipose tissue; LAD, left atrial diameter; LDL-C, low-density lipoprotein cholesterol; NT-proBNP, N-terminal pro-B-type natriuretic peptide.

In patients with sleep apnea, the AUC values for sex, age, BMI, AHI, NT-proBNP, LDL-C, LAD, and EAT relative volume were 0.65 (0.57–0.74), 0.74 (0.66–0.82), 0.65 (0.56–0.74), 0.74 (0.66–0.82), 0.97 (0.94–1.00), 0.41 (0.32–0.50), 0.77 (0.71–0.85), and 0.74 (0.66–0.82), respectively (*p* < 0.05). Among these variables, NT-proBNP demonstrated the highest predictive efficacy (best cutoff: 641) (Figure 3).

### 3.5 Diagnostic efficacy of predictive models for HFpEF in patients with SA

The diagnostic performance of the four established models in patients with SA cohort was assessed using ROC curve analysis. Compared with random guessing, all models demonstrated statistically significant discriminatory ability (all *P* < 0.001). The area under the curve (AUC) for Model 1, which included gender and LAD, was 0.847 (95% CI: 0.789–0.906). Model 2, comprising gender and EAT relative volume, achieved an AUC of 0.798 (95% CI: 0.730–0.866). Model 3, which combined these structural parameters, achieved an AUC of 0.893 (95% CI: 0.843–0.942). Notably, Model 4, which included gender, LAD, EAT relative volume, and NT-proBNP, demonstrated the highest diagnostic performance, yielding an AUC of 0.971 (95% CI: 0.942–1.000). The C-statistic of the model containing only NT-proBNP was 0.942 (95% CI: 0.901–0.983). After the addition of gender, LAD, and relative EAT volume to the model, the C-statistic increased to 0.971 (95% CI: 0.942–1.000), with a statistically significant difference (*P* = 0.028). The continuous NRI was 0.682 (95% CI: 0.415–0.949, *P* < 0.001), and the IDI was 0.124 (95% CI: 0.076–0.172, *P* < 0.001), indicating that the addition of LAD and EAT significantly improved the reclassification and discriminatory ability of the model.

**Figure 4:**
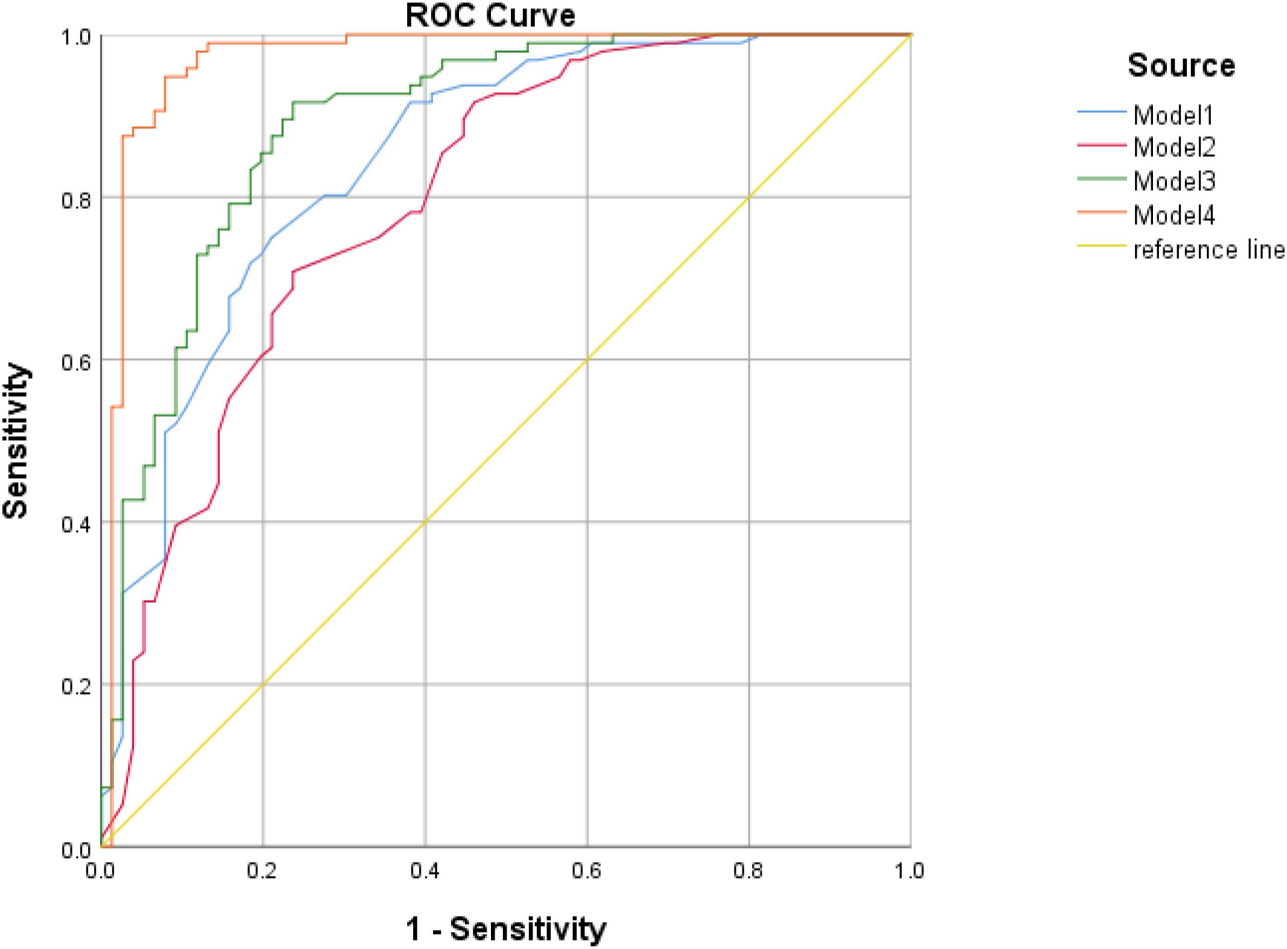
Receiver operating characteristic (ROC) curves for the four predictive models for HFpEF in patients with SA. Model 1 included sex and left atrial diameter (LAD). Model 2 included sex and epicardial adipose tissue (EAT) relative volume. Model 3 combined sex, LAD, and EAT relative volume. Model 4 comprised sex, LAD, EAT relative volume, and N-terminal pro-B-type natriuretic peptide (NT-proBNP). The diagonal reference line indicates an area under the curve (AUC) of 0.5.

This stepwise improvement suggests that combining biomarkers with cardiac structural parameters may produce synergistic effects, thereby potentially improving the accuracy of HFpEF identification. Nevertheless, these preliminary results should be interpreted with caution; future validation in larger, independent external cohorts is required to confirm the superiority of this comprehensive approach (Figure 4).

### 3.6 Cox regression analysis of poor prognosis in patients with SA combined with HFpEF

All indicators were categorized into high- and low-value groups based on optimal cutoff values from the ROC curve.

In the univariate Cox regression analysis, NT-proBNP level, BMI, AHI, and LAD (*P* < 0.05) were identified as risk factors for poor prognosis in patients with SA and HFpEF. Multivariate Cox regression revealed that NT-proBNP remained an independent risk factor for poor prognosis in SA patients with HFpEF (Table 5).

**Table 5:** Cox regression analysis of poor prognosis in patients with SA combined with HFpEF.

Group	Univariate logistic			Multivariate logistic		
	*HR*	95% CI	*P*	*HR*	95% CI	*P*
NT-proBNP	2.15	1.43–3.24	0.000	1.89	1.24–2.88	0.003
BMI	1.21	1.01–1.87	0.046			
AHI	2.08	1.00–4.32	0.049			
LAD	1.09	1.03–1.15	0.013			
EAT relative volume	1.34	0.58–3.05	0.491			

BMI, body mass index; NT-proBNP, N-terminal brain natriuretic peptide precursor; AHI, apnea–hypopnea index; LAD, left atrial diameter; EAT relative volume, epicardial adipose tissue relative volume.

## 4 Discussion

The present study systematically investigated the associations of LAD and EAT with sleep apnea (SA) and heart failure with preserved ejection fraction (HFpEF). Our findings suggest that both LAD and relative EAT volume were significantly associated with the presence of HFpEF in patients with SA, suggesting that routine assessment of these two parameters may facilitate the early identification of SA patients at increased risk of developing HFpEF.

### 4.1 Central role and clinical significance of LAD in the SA–HFpEF interaction

Structural and functional impairment of the LA are widely recognized as independent predictors of morbidity and mortality in patients with HFpEF, and their pathophysiological significance has been extensively documented (10, 11). In our cohort, LAD was significantly greater in the HFpEF group than in the non-HFpEF group. Moreover, multivariate logistic regression analysis identified LAD as an independent factor associated with HFpEF in SA patients, which is consistent with previous findings. Donal et al. (12) reported that LA enlargement is primarily driven by increased LV filling pressure and impaired diastolic function, both of which are pivotal for the diagnosis and prognostic evaluation of HFpEF. In patients with SA, LAD has been shown to increase with the severity of apnea episodes (13, 14). The underlying mechanism likely involves recurrent intermittent hypoxia, which impairs ventricular diastolic function while simultaneously activating the sympathetic nervous system and suppressing parasympathetic activity. In addition, intermittent hypoxia triggers systemic inflammation and oxidative stress, both of which further exacerbate atrial structural and electrical remodeling (15). These pathological alterations may ultimately accelerate the progression to HFpEF.

As LV end-diastolic pressure rises, the LA undergoes progressive dilation and eventual decompensation, leading to reduced atrial reserve and contractile function, which may further exacerbate the progression of heart failure (16). Our correlation analysis revealed a significant positive association between LAD and NT-proBNP levels, consistent with the findings of Turkoglu and Kircicegi Cicekdag et al. (17). This reinforces the notion that LA enlargement may serve as a surrogate marker of increased cardiac pressure load and neurohormonal activation. Given that LA enlargement reflects increased myocardial stiffness and elevated filling pressures (18–21), it may create a “vicious cycle” of structural and functional deterioration. This ultimately leads to cardiac remodeling and the development of HFpEF, which is consistent with the findings of several clinical studies indicating that “the LAD is significantly larger in patients with chronic heart failure than in those without heart failure” (22, 23). Consequently, incorporating LAD into the routine evaluation of SA patients may be useful for early detection of cardiac remodeling and improved risk stratification.

It is noteworthy that in our study, although patients in the non-HFpEF group did not meet the full clinical criteria for heart failure, some exhibited mildly elevated NT-proBNP levels. This increase may be attributed to the high prevalence of obesity (as indicated by BMI) and systemic hypertension in patients with SA, both of which may induce subclinical cardiac burden prior to the clinical onset of HFpEF. These findings further underscore the potential clinical value of LAD and EAT in identifying early cardiovascular risk in this vulnerable population.

### 4.2 EAT as a metabolic inflammatory mediator linking SA and HFpEF

EAT is a unique visceral fat depot located between the myocardium and the visceral pericardium. Excessive EAT accumulation may contribute to HFpEF through both mechanical and metabolic pathways. Mechanically, EAT can exert paracardial compression, increasing cardiac load and promoting compensatory remodeling. Metabolically, EAT acts as an active endocrine organ, secreting a range of pro-inflammatory adipokines and mediators.

Pathological accumulation of EAT may induce local inflammation, oxidative stress, and endothelial dysfunction, thereby potentially impairing myocardial microcirculation and diastolic filling. These processes may culminate in LA enlargement and myocardial fibrosis (24–26). Supporting this hypothesis, Wu et al. (27) and Jin et al. (28) observed significantly higher EAT volumes in HFpEF patients compared with healthy controls, with increased EAT correlating with impaired LA function. Furthermore, Parisi et al. (29) found that heart failure patients with comorbid SA exhibited greater EAT thickness than those without SA, suggesting that SA-induced sympathetic activation and systemic inflammation may specifically drive EAT expansion.

One proposed molecular mechanism involves the activation of the interleukin-33 (IL-33)/suppression of tumorigenicity-2 signaling pathway, which may promote myocardial fibrosis and LA remodeling (17, 20, 30, 31). Additionally, EAT thickness correlates with sympathetic hyperactivity and may serve as a local source of catecholamines, suggesting that sympathetic overactivation is a key pathophysiological bridge between SA and EAT accumulation (15, 32). Notably, CPAP therapy has been shown to reduce EAT thickness and improve metabolic profiles in SA patients (33), marking EAT as a potential therapeutic target for this patient population. Despite its potential mechanistic and prognostic relevance, EAT quantification by cardiac CT may not yet be suitable for universal routine screening due to cost, radiation exposure, and the lack of standardized acquisition protocols. At present, EAT assessment may be more appropriate for selected high-risk patients who are already undergoing cardiac imaging. Simpler surrogate markers and echocardiographic approaches may improve future clinical applicability.

### 4.3 Clinical implications and future perspectives

With the rising global prevalence of HFpEF, optimizing risk stratification and clinical management has become a top priority in cardiovascular research. To the best of our knowledge, the present study is among the first to systematically evaluate the combined association of LAD and EAT with HFpEF in patients with SA. Our findings highlight that both parameters are independently associated with the condition and closely related to disease severity in this patient population.

In clinical practice, intensified monitoring of LAD and EAT in SA patients is warranted. When integrated with established biomarkers such as NT-proBNP, these imaging metrics could help refine risk stratification and facilitate personalized management strategies. From a therapeutic standpoint, emerging agents such as SGLT2 inhibitors and GLP-1 receptor agonists—known for their beneficial effects on cardiac metabolism and for reducing inflammation—may potentially mitigate EAT accumulation and adverse LA remodeling (34, 35). Future research should investigate whether early pharmacological intervention targeting these pathways can alter the clinical trajectory of patients with both SA and HFpEF.

## 5 Limitations

This study has several limitations that should be acknowledged. First, because NT-proBNP formed part of the diagnostic framework for HFpEF and was simultaneously analyzed as a predictor, incorporation bias cannot be excluded. Therefore, the predictive performance of NT-proBNP and related multivariable models should be interpreted cautiously. Second, this was a single-center observational study with a relatively small sample size, which may limit the generalizability of our findings to other populations. Third, although HFpEF was diagnosed using a standardized multiparametric framework, we were unable to measure several key echocardiographic parameters (e.g., E/e′ ratio, left atrial volume index, and tricuspid regurgitation velocity) due to institutional limitations in echocardiographic assessment. Fourth, this study was a single-center observational study, and all patients included were inpatients. Consequently, our findings may not be generalizable to outpatients or to patients from different ethnic groups or geographical regions. No patients received CPAP therapy during the study period, which enabled us to exclude the confounding effects of CPAP on cardiac remodeling and EAT accumulation and to assess the independent relationship between SA itself and HFpEF. However, this also implies that our findings may not apply to patients with SA who are receiving active CPAP therapy. Future multicenter studies involving a diverse patient population are required to validate our findings. Finally, the potential risk of statistical overfitting must be acknowledged, as the predictive model was not externally validated. Given the ratio of candidate predictors to event incidence, the significantly high AUC observed for the combined model should be interpreted with caution. These preliminary findings require further validation through prospective multicenter trials to establish the long-term robustness and clinical generalizability of the proposed predictive markers.

## Data Availability

The raw data supporting the conclusions of this article will be made available by the authors, without undue reservation.
